# Conventional and Non-Conventional Dysplasias Associated with Inflammatory Bowel Disease—A Single-Centre Experience

**DOI:** 10.3390/medsci14010078

**Published:** 2026-02-10

**Authors:** Anita Sejben, Zsófia Balajthy, Zsófia Krisztina Török, Szintia Almási, Tamás Lantos

**Affiliations:** 1 Department of Pathology, University of Szeged, 6725 Szeged, Hungary; 2 Department of Medical Physics and Informatics, University of Szeged, 6720 Szeged, Hungary

**Keywords:** inflammatory bowel disease, ulcerative colitis, Crohn’s disease, non-conventional dysplasia, IBD-associated neoplasm

## Abstract

**Background**: Recent studies have identified multiple subtypes of non-conventional dysplastic lesions associated with inflammatory bowel disease (IBD). This study aimed to characterise and compare all IBD-associated dysplasias and determine their prevalence within a Southern Hungarian population. **Methods**: A consecutive cohort of IBD patients between 2011 and 2023 was retrospectively analysed. All available hyper- or neoplastic samples were reclassified according to current pathological criteria. **Results**: In this 13-year retrospective, single-centre, cohort study, 2396 IBD patients were identified, with 176 possessing samples for re-evaluation. Conventional dysplasia was identified in 130 patients (74%), non-conventional dysplasia in 50 patients (28%), and 33 patients were diagnosed with both (19%). Age (OR = 1.06; 95% CI: [1.03–1.09]; *p* < 0.001) and male gender (OR = 2.63; 95% CI: [1.07–6.45]; *p* = 0.032) were associated with higher odds of conventional dysplasia, compared with non-conventional dysplasia. Colorectal carcinoma development and M stage were associated with lower odds of conventional dysplasia: OR = 0.17 ([0.06–0.47]; *p* < 0.001) and OR = 0.05 ([0.01–0.5]; *p* = 0.006), respectively. Significant associations were observed with macroscopic appearance (*p* = 0.013), grade (*p* = 0.002), localisation (*p* < 0.001), size (*p* = 0.016), macroscopic morphology (*p* = 0.004), grade (*p* < 0.001), histological subtype (*p* = 0.001), T stage (*p* = 0.008) and microsatellite status (*p* = 0.019). **Conclusions**: Non-conventional dysplasias were identified in a substantial proportion of neoplastic lesions (50/176; 28%) among IBD patients. These findings highlight the importance of thorough histopathological evaluation to confirm complete resection. Patients with IBD-associated dysplasia, particularly non-conventional types, may benefit from intensified surveillance strategies.

## 1. Introduction

Inflammatory bowel disease (IBD) represents a group of chronic, idiopathic gastrointestinal diseases, characterised by episodes of relapsing interstitial inflammation, driven by inappropriate immune responses to an altered gut microbiome in genetically susceptible individuals. The two main subtypes of IBD are ulcerative colitis (UC) and Crohn’s disease (CD). A third diagnostic category, indeterminate colitis (IBD-U), is used for cases of IBD in which a definitive diagnosis of UC or CD cannot be established based on histopathological and clinical data [1].

Patients with IBD are at an increased risk of developing dysplasia and colorectal cancer (CRC) [2,3,4]. The pathogenesis of IBD-associated CRC is believed to result from chronic, sustained inflammation of the colorectal mucosa, compounded by genetic, immunological, microbial, and environmental factors [5]. Several risk factors have been identified that contribute to neoplastic progression in IBD, including the duration, severity, and anatomical extent of the disease. Additionally, male sex and the presence of primary sclerosing cholangitis (PSC) have been associated with an elevated risk [6,7].

Current evidence suggests that individuals with IBD have a 1.2- to 2-fold increased risk of developing colorectal neoplasia compared to the general population [6]. Both UC and CD carry a comparable risk for dysplasia and CRC; however, the risk is higher in patients with UC, and it is important to note that neoplastic lesions in IBD tend to occur at a younger age than sporadic cases. Nevertheless, advancements in endoscopic surveillance and early therapeutic interventions have contributed to a reduction in CRC-related mortality and an improvement in overall survival among IBD patients [8,9].

The conventional form of dysplasia in the gastrointestinal tract is defined by cytological features of intestinal differentiation and characteristics similar to those of conventional colonic adenomas [10]. In addition to conventional dysplasia, several distinct subtypes of non-conventional dysplasia have recently been identified in the context of IBD. These lesions are characterised by atypical morphological features that do not resemble traditional adenomatous dysplasia [11]. To date, seven non-conventional dysplasia subtypes have been described: hypermucinous dysplasia, goblet cell-deficient dysplasia (GCD), dysplasia with increased Paneth cell differentiation (DPD), crypt cell dysplasia (CCD), traditional serrated adenoma (TSA)-like dysplasia, sessile serrated lesion (SSL)-like dysplasia, and serrated dysplasia not otherwise specified (NOS). Currently, strict morphological criteria for the identification of these subtypes have not been fully established. As a result, these lesions may be misinterpreted as benign or reactive changes, particularly in routine diagnostic practice, posing a challenge for accurate histopathological recognition and diagnosis. Recognition of non-conventional dysplasia subtypes is clinically important, as several of these lesions appear to carry a higher risk of progression to high-grade dysplasia or CRC. This elevated risk is likely due, in part, to the frequent presence of aneuploidy observed in these lesions. Moreover, non-conventional dysplasias are more often associated with poorly differentiated adenocarcinomas and signet ring cell carcinomas, further suggesting a more aggressive biological behaviour [12,13,14,15,16,17,18].

Certain subtypes, such as CCD and serrated dysplasia NOS, are frequently detected as flat lesions on endoscopy, making them more challenging to identify during routine surveillance. Collectively, these characteristics suggest that non-conventional dysplasias may be associated with a poorer prognosis compared to conventional dysplastic subtypes [12,13,14,15,16,17,18].

This retrospective, cohort study aimed to comprehensively examine both conventional and non-conventional dysplasia subtypes associated with IBD, to determine their prevalence within a Southern Hungarian population, and to identify clinicopathological features with potential prognostic significance.

## 2. Materials and Methods

### 2.1. Study Design

A retrospective database was established, covering the period between 2011 and 2023, and included all patients diagnosed with and/or treated for IBD at the Albert Szent-Györgyi Clinical Centre, University of Szeged. All patients with histologically confirmed hyper- or neoplastic lesions were subsequently identified, and their biopsy and surgical resection samples were subjected to detailed histopathological re-evaluation. Clinical data recorded for each patient included age, gender, ethnicity, IBD subtype—classified as UC, CD, or IBD-U—and the duration and extent of IBD (the latter based on earlier histopathological reports).

### 2.2. Histopathological Evaluation

All available hyper- and neoplastic samples, including biopsies and CRC resection specimens, were retrospectively reviewed by a fellowship-trained gastrointestinal pathologist (A.S.) to reassess and recategorise all lesions. Dysplasia was classified as either conventional or non-conventional, based on the dominant histological pattern observed in at least 50% of the tissue sample. Conventional dysplasias were defined as tubular adenomas (TAs), tubulovillous adenomas (TVAs), or villous adenomas (VAs). Non-conventional dysplasias were categorised according to the criteria proposed by Choi et al. and included hypermucinous dysplasia, GCD, DPD, CCD, TSA-like dysplasia, SSL-like dysplasia, and serrated dysplasia NOS [12].

For all included cases, detailed histological information was collected, including the date of diagnosis, anatomical location of the lesion, lesion size in centimetres, macroscopic appearance (flat or polypoid), and histological subtype. In patients who developed CRC during the follow-up period, additional pathological data were recorded. These included tumour location, size, macroscopic appearance, histological grade, and histological subtype. Subtypes were assessed using both the current World Health Organisation (WHO) classification and recently recognised patterns, such as serrated and goblet cell-deficient adenocarcinomas [19,20]. Tumour staging was recorded according to the TNM classification system, and microsatellite status was evaluated using immunohistochemistry, in accordance with established protocols and prior literature.

### 2.3. Statistical Analyses

Statistical analyses were conducted using SPSS Statistics version 23.0 (IBM Corp., Armonk, NY, USA). Group comparisons were performed using the Chi-square test, Mann–Whitney U test, and Kruskal–Wallis test, where appropriate. In such cases, where applicable, univariable logistic regression analyses were carried out to evaluate the strength and direction of associations, calculating odds ratios (ORs) and their 95% confidence intervals (95% CIs). A *p*-value of less than 0.05 was considered statistically significant.

### 2.4. Ethical Approval

This study was reviewed and approved by the Hungarian Medical Research Council (BM/28834-1/2024) and the Regional and Institutional Human Biomedical Research Ethics Committee of the University of Szeged (5670) and was conducted in accordance with relevant ethical guidelines.

## 3. Results

### 3.1. General Data

Over the 13 years of the study, 2396 patients were diagnosed with and/or treated for IBD at the University of Szeged. The majority of patients resided in Southern Hungary (statistical region: the Southern Great Plain) and neighbouring regions of Romania, and all were of Caucasian ethnicity. From this cohort, 176 patients (7.3%) were identified as having an IBD-associated hyper- or neoplastic lesion and were included in further analysis. Among these 176 patients, male predominance (60%) was observed (male-to-female ratio: 106:70). The mean age at the end of the follow-up period was 61.2 years (median: 63 years; range: 28–86 years). The majority of the total IBD population was diagnosed with UC (*n* = 1400; 58.4%), followed by CD (*n* = 970; 40.5%), while a smaller subset (*n* = 26; 1.1%) of cases was classified as IBD-U. Neoplastic lesions were most frequently located in the left colon (*n* = 90; 52.9%), in alignment with the predominant localisation of IBD, followed by cases involving pancolitis (*n* = 46; 26.1%).

### 3.2. Examination of IBD-Associated Conventional Dysplasia Cases

**Figure 1:**
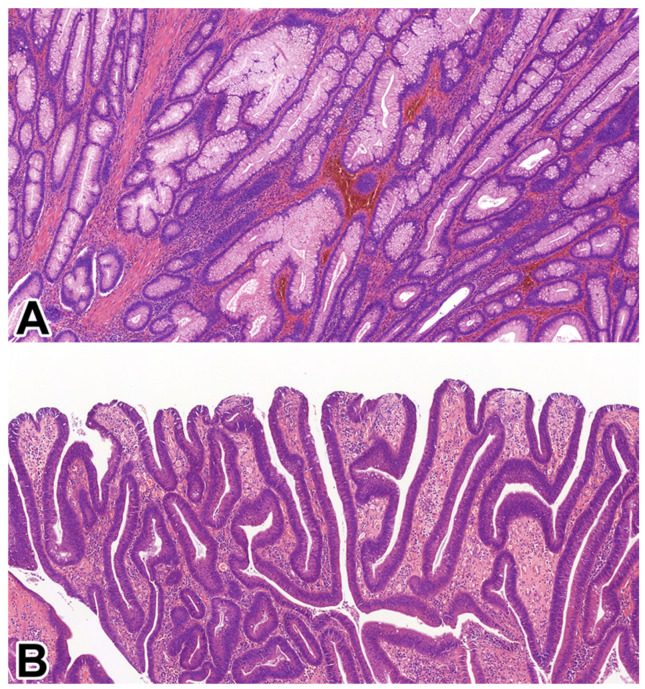
Microscopic appearance of IBD-associated, conventional dysplasias. (**A**) IBD-associated TA with low-grade dysplasia (HE, 5×). (**B**) IBD-associated TVA with low-grade dysplasia (HE, 5×). Abbreviations: IBD—inflammatory bowel disease, HE—haematoxylin and eosin, TA—tubular adenoma, TVA—tubulovillous adenoma.

Conventional dysplasia was identified in 130 cases, representing 5.4% of the total IBD patient population. The majority of these lesions were TAs (*n* = 106; 81.5%), followed by TVAs (*n* = 24; 18.5%). Notably, no VAs were detected in this cohort. Representative histological features of conventional IBD-associated dysplasias are shown in Figure 1, and associated clinicopathological characteristics are summarised in Table 1. The mean age of patients with conventional dysplasia was 52.8 years (median: 50.5; range: 29–83), with a male-to-female ratio of 32:18. Most conventional dysplastic lesions were associated with UC (*n* = 41; 82%). Regarding disease distribution, left-sided colitis was the most common IBD localisation (*n* = 58; 44.6%), followed by both pancolitis (*n* = 36; 27.6%) and right-sided colitis (*n* = 36; 27.8%). The mean duration of IBD before dysplasia diagnosis was 14 years (median: 13; range: 1–47). Lesions were predominantly located in the left colon (*n* = 88; 67.7%), with an average lesion size of 1.2 cm (median: 1.0 cm; range: 0.1–2.5 cm). On endoscopy, conventional dysplasias were most often polypoid in appearance (*n* = 106; 81.5%) and histologically classified as low-grade (*n* = 117; 90%).

During the follow-up period, 26 of the 130 patients with conventional dysplasia (20%) developed CRC. More than half of these cancers (*n* = 14; 53.8%) were localised to the left colon. The vast majority of tumours were histologically low-grade (grade 1 or 2; *n* = 22; 91.7%). Conventional adenocarcinoma was the most frequently observed histological subtype (*n* = 18; 69.3%), followed by mucinous (*n* = 5; 19.2%), medullary (*n* = 1; 3.8%), signet ring cell (*n* = 1; 3.8%), and goblet cell-deficient adenocarcinoma (*n* = 1; 3.8%). The mean maximum tumour diameter was 3.7 cm (median: 1.0 cm; range: 0.2–8.4 cm), and 15 tumours (57.7%) were macroscopically polypoid. Tumour staging revealed T1 lesions in four cases (15.4%), T2 in two (7.7%), T3 in nine (34.6%), and T4 in eight cases (42.3%). Lymph node involvement included N1 status in six cases (23%) and N2 in two cases (7.7%). Metastatic disease (M1 stage) was present in six patients (23%), and lymphovascular invasion was confirmed in nine cases (34.6%). Microsatellite instability (MSI) testing by immunohistochemistry was performed in 19 cases, with mismatch repair (MMR) deficiency detected in 5 patients (26.3%). Age (per one year increase), male gender and dysplasia size (per 1 cm increase) were associated with higher odds for developing conventional dysplasia: OR = 1.06 (95% CI: [1.03–1.09]; *p* < 0.001), OR = 2.46 (95% CI: [1.24–4.88]; *p* = 0.009) and OR = 1.27 (95% CI: [1.12–1.45]; *p* < 0.001), respectively. Conversely, CRC development and presence of distant metastases were associated with lower odds for the development of conventional dysplasia: OR = 0.33 (95% CI: [0.16–0.67]; *p* = 0.002) and OR = 0.23 (95% CI: [0.08–0.7]; *p* = 0.01), respectively.

Additional significant associations were observed with the macroscopic appearance (*p* < 0.001), dysplasia grade (*p* < 0.001), localisation (*p* < 0.001), macroscopic morphology (*p* = 0.013), CRC grade (*p* < 0.001), tumour localisation (*p* = 0.004), histological subtype (*p* = 0.006) and pT stage (*p* = 0.005).

### 3.3. Examination of IBD-Associated Non-Conventional Dysplasia Cases

**Figure 2:**
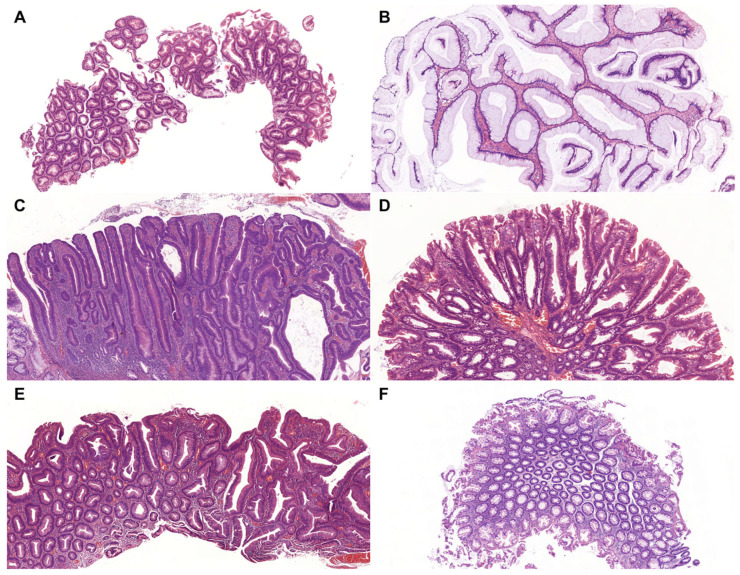
Microscopic appearance of IBD-associated, non-conventional dysplasias and SEC. (**A**) Serrated dysplasia, NOS (HE, 5×). (**B**) Hypermucinous dysplasia (HE, 5×). (**C**) GCD (HE, 5×). (**D**) SSL-like dysplasia (HE, 5×). (**E**) TSA-like dysplasia (HE, 5×). (**F**) SEC (HE, 5×). Abbreviations: GCD—goblet cell-deficient dysplasia, IBD—inflammatory bowel disease, HE—haematoxylin and eosin, NOS—not otherwise specified, SEC—serrated epithelial change, SSL—sessile serrated lesion, TSA—traditional serrated adenoma.

Non-conventional dysplasia was identified in 50 patients, accounting for 2.1% of the total IBD cohort (2396 patients). The most frequently observed histological subtype was serrated dysplasia NOS (*n* = 20; 40%), followed by hypermucinous (*n* = 14; 28%), GCD (*n* = 9; 18%), SSL-like (*n* = 6; 12%), and TSA-like dysplasia (*n* = 1; 2%). Representative histological features of non-conventional dysplasias are shown in Figure 2, and detailed clinicopathological data are summarised in Table 2. The mean age at diagnosis for patients with non-conventional dysplasia was 61.2 years (median: 63; range: 28–94), with a male-to-female ratio of 28:22. The majority of lesions arose in the context of UC (*n* = 42; 84%). Regarding disease extent, left-sided colitis was the most prevalent IBD subtype (*n* = 29; 58%), followed by pancolitis (*n* = 20; 40%) and right-sided colitis (*n* = 1; 2%). The mean duration of IBD before dysplasia diagnosis was 14 years (median: 14; range: 2–58). In accordance with the underlying IBD distribution, most dysplastic lesions were located in the left colon (*n* = 32; 64%). The average lesion size was 0.7 cm (median: 0.5 cm; range: 0.2–2.1 cm), and endoscopic appearance was predominantly polypoid (*n* = 35; 70%). The vast majority of lesions were classified as low-grade dysplasia (*n* = 49; 98%). Serrated epithelial changes (SECs) were noted in five cases.

Among the 50 patients with non-conventional dysplasia, 18 cases (36%) progressed to CRC. The majority of CRCs were localised to the left colon (*n* = 12; 66.7%) and were histologically low-grade (*n* = 14; 77.8%). Conventional adenocarcinoma was the most prevalent histological subtype (*n* = 11; 61.1%), followed by mucinous (*n* = 4; 22.2%), signet ring cell (*n* = 2; 11.1%), and serrated adenocarcinoma (*n* = 1; 5.6%). The mean tumour size was 3.1 cm (median: 3.5 cm; range: 0.2–6.5 cm). Half of the tumours presented as polypoid masses (*n* = 9; 50%), while the other half exhibited an infiltrative morphology. Tumour staging revealed T1 in five cases (27.8%), T2 in three (16.7%), T3 in six (33.3%), and T4 in four patients (22.2%). Nodal involvement was recorded in five patients: N1 in two cases (11.1%) and N2 in three cases (16.7%), with no nodal metastases in the remaining patients. Distant metastases (M1) were already present at diagnosis in five cases (27.8%), and lymphovascular invasion was confirmed in five cases (27.8%). MSI testing by immunohistochemistry was performed in 14 cases, with MMR deficiency identified in 2 patients (14%). Age (per one-year increase), IBD subtype (CD vs. UC), CRC development, CRC multifocality, M stage, and the presence of lymphovascular invasion were associated with higher odds for developing non-conventional dysplasia: OR = 0.97 (95% CI: [0.95–0.9]; *p* = 0.003), OR = 0.43 (95% CI: [0.19–0.96]; *p* = 0.037), OR = 3.97 (95% CI: [1.95–8.07]; *p* < 0.001), OR = 4.82 (95% CI: [1.89–12.34]; *p* < 0.001), OR = 4.54 (95% CI: [1.44–14.27; *p* = 0.012), OR = 3.19 (95% CI: [1.12–9.06]; *p* = 0.024), respectively. Additional significant associations were found between non-conventional dysplasia and macroscopic appearance of the dysplastic lesion (*p* = 0.011), CRC localisation (*p* < 0.001), size (*p* < 0.001), macroscopic morphology (*p* < 0.001), grade (*p* < 0.001), histological subtype (*p* < 0.001), and pT stage (*p* = 0.003).

### 3.4. Examination of IBD-Associated Combined Conventional and Non-Conventional Dysplasia Cases

A total of 33 patients (1.4% of all IBD cases) were identified with both conventional and non-conventional dysplasia. The mean age of this subgroup was 61.3 years (median: 63; range: 40–83), with a marked male predominance (male-to-female ratio: 24:9). The majority of these patients were diagnosed with UC (*n* = 26, 78.8%), with an average IBD duration of 14.2 years before dysplasia diagnosis (median: 13; range: 3–40). Disease distribution predominantly involved the left colon (*n* = 18; 54.5%), followed by pancolitis (*n* = 11; 33.0%). Correspondingly, the dysplastic lesions were mainly localised to the left colon (*n* = 24; 72.7%), with an average lesion size of 1.0 cm (median: 1; range: 0.2–2.5 cm). The majority of dominant lesions exhibited a polypoid morphology on endoscopy (*n* = 19; 57.6%). The most frequently observed histological combination was serrated dysplasia NOS with TA (*n* = 3; 6%). CRC developed in six patients (18.2%) within this subgroup. Most of these tumours were localised to the left colon (*n* = 5; 83.3%) and had an average size of 3.1 cm (median: 2.0; range: 0.7–7.5 cm). Half of the tumours (*n* = 3; 50%) exhibited an infiltrative macroscopic morphology, and most were histologically low-grade (*n* = 4; 66.7%). Conventional adenocarcinoma was the predominant histological pattern (*n* = 3; 50%), followed by mucinous (*n* = 2; 33.3%) and signet ring cell carcinoma (*n* = 1; 16.7%). Notably, five of the six tumours (83.3%) demonstrated mixed histological features within the same lesion. Advanced tumour stages were common, with four cases (66.7%) diagnosed at T3 or T4. Lymphovascular invasion was detected in three cases (50%), and distant metastasis was observed in two patients (33.3%) during follow-up. Dysplasia size, CRC development and multifocality were associated with higher odds for developing combined conventional and non-conventional dysplasias: OR = 1.14 (95% CI: [1.05–1.23]; *p* = 0.002), OR = 3.01 (95% CI: [1.36–6.65]; *p* = 0.005), and OR = 6 (95% CI: [2.32–15.51]; *p* < 0.001), respectively. Additional significant associations were found between the co-occurrence of conventional and non-conventional dysplasia and IBD duration (*p* = 0.01), CRC localisation (*p* = 0.012), size (*p* = 0.003), macroscopic appearance (*p* < 0.001), histological grade (*p* < 0.001) and subtype (*p* = 0.024), T (*p* = 0.007), and N stage (*p* = 0.025).

### 3.5. Comparison of the Exclusively Conventional and Non-Conventional Dysplasia Cohorts

Exclusively conventional dysplasia was identified in 97 cases (4% of all IBD cases), whereas exclusively non-conventional dysplasia was observed in 17 (0.7% of all IBD cases). Age (per one-year increase) and male gender were associated with higher odds of conventional dysplasia (compared with non-conventional dysplasia): OR = 1.06 (95% CI: [1.03–1.09]; *p* < 0.001) and OR = 2.63 (95% CI: [1.07–6.45]; *p* = 0.032), respectively. Conversely, colorectal carcinoma development and M stage were associated with lower odds of conventional dysplasia (compared with non-conventional dysplasia): OR = 0.17 (95% CI: [0.06–0.47]; *p* < 0.001) and OR = 0.05 (95% CI: [0.01–0.5]; *p* = 0.006), respectively. Moreover, significant associations were observed between the conventional versus non-conventional dysplasia population regarding macroscopic appearance (*p* = 0.013), dysplasia grade (*p* = 0.002), localisation (*p* < 0.001), size (*p* = 0.016), macroscopic morphology (*p* = 0.004), CRC grade (*p* < 0.001), histological subtype (*p* = 0.001), T stage (*p* = 0.008) and microsatellite status (*p* = 0.019).

## 4. Discussion

For decades, IBD-associated dysplasias were diagnosed and graded using the Riddell criteria, which focused primarily on histological assessment. However, a paradigm shift occurred following the publication of the SCENIC consensus guidelines in 2015, which introduced a new dimension to dysplasia classification by emphasising endoscopic visibility—distinguishing between visible (polypoid) and invisible dysplasia rather than relying solely on histological features. This shift also eliminated the need to distinguish IBD-associated polypoid dysplasias from sporadic adenomas for therapeutic decision-making, as endoscopic resection is now generally sufficient for both lesion types [13,21,22]. Another significant development in the field was the introduction of the IBD-associated non-conventional dysplasia classification by Choi et al. in 2020 [12]. This new framework encompasses all dysplasia subtypes that deviate histologically or clinicopathologically from traditional non-IBD conventional dysplasias. Although the characteristics and molecular underpinnings of these lesions remain incompletely understood, accumulating evidence suggests that several subtypes are associated with a higher risk of progression to CRC. Consequently, the early recognition and accurate diagnosis of non-conventional dysplasias are considered critical in IBD surveillance and patient management [12].

In this retrospective cohort study, all hyper- and neoplastic lesions from IBD patients treated at the University of Szeged between 2011 and 2023 were re-evaluated, with particular emphasis on identifying non-conventional dysplasias based on the most recent classification system. Several similarities and differences were observed between conventional and non-conventional dysplasias. The discrepancy in prevalence—5.4% for conventional and 2.1% for non-conventional dysplasias—may, in part, reflect the inclusion of cases diagnosed before the recognition and widespread adoption of the IBD-associated non-conventional dysplasia classification. Additionally, non-conventional dysplasia subtypes are inherently more heterogeneous and less well-defined histologically, which may have contributed to underdiagnosis in earlier years.

Patients with non-conventional dysplasia were generally older at the time of diagnosis, with a mean age of 65.5 years compared to 52.3 years in the conventional dysplasia group. However, it is important to note that random biopsies may not have been consistently obtained in earlier years of the cohort. In the conventional dysplasia group, female predominance was noted, while non-conventional dysplasia was associated with male gender. It has to be emphasised that dysplasia was associated with UC in both groups, with a predominance of left-sided disease. The duration of IBD before dysplasia diagnosis did not differ significantly between the two groups. Even though a significant association was found between the endoscopic morphology of the non-conventional dysplasia subgroup (polypoid appearance was noted in 64% of the conventional group and 86% of the non-conventional group), this may also be influenced by detection biases or sampling protocols over time.

CRC developed more frequently in patients with non-conventional dysplasia (90%) compared to those with conventional dysplasia (60%). However, conventional dysplasias were more often associated with mucinous adenocarcinomas. Based on UC, dysplastic lesions and CRCs were predominantly localised to the left colon in both groups. CRCs associated with non-conventional dysplasias were macroscopically larger (median: 5.5 cm vs. 3.5 cm). Pathological stage 2 or 3 CRC was more commonly observed in association with conventional dysplasias (24% vs. 4%). Distant metastases (M1 stage) were also more commonly observed in patients with non-conventional dysplasia (99% vs. 84%).

Our retrospective, consecutive, cohort study represents a continuation of our former IBD-associated dysplasia studies, in an expanded cohort [23]. To our knowledge, this is the first consecutive analysis conducted on the Hungarian IBD population, and we emphasise that no similar comprehensive, centre-wide examination of all IBD patients at a gastroenterology centre has yet been reported in the literature; therefore, the prevalence of NCDs is difficult to ascertain. Within this consecutive setting, non-conventional dysplasias were identified in a relatively substantial proportion of patients, comprising 2.1% of all IBD cases and 28.4% of all IBD-associated neoplastic lesions. Interestingly, our cohort did not include cases of CCD or DPD, which may reflect underlying population-specific differences. Nonetheless, the hypermucinous subtype was consistently identified as the second most common non-conventional dysplasia in both North American and Hungarian cohorts [17].

One notable limitation of our study lies in the extended timeframe of data collection, spanning over a decade. The classification of IBD-associated non-conventional dysplasia was only introduced in 2020, and as such, earlier endoscopic and histopathologic evaluations may not have recognised or recorded these lesions accurately. This may have resulted in underdiagnosis or misclassification in the earlier years of the study period. Moreover, only samples with a prior histological diagnosis of dysplasia or neoplasia were re-evaluated. Consequently, cases in which no initial suspicion for neoplastic change was raised were excluded from reassessment, potentially leading to the omission of additional relevant dysplastic lesions. Furthermore, we were not able to access data that might influence carcinogenesis, including smoking status and first-degree family history of colorectal cancer.

Given the current limited understanding of the molecular pathogenesis of non-conventional dysplasias, further genetic characterisation of these lesions remains a priority in our ongoing research.

## 5. Conclusions

This study provides the first comprehensive, consecutive assessment of IBD-associated neoplastic lesions in a Hungarian tertiary centre, offering a robust baseline for future national comparisons. By systematically reclassifying lesions using updated criteria, it highlights the diagnostic advantages of contemporary terminology for capturing clinically significant non-conventional dysplasia. The substantial proportion of non-conventional dysplasia identified demonstrates that these entities are not rare and can be recognised reliably in routine practice. Their distinct clinicopathological profile offers clinicians an opportunity to identify higher-risk patients earlier and adapt surveillance accordingly. The observed association between non-conventional dysplasia and aggressive carcinoma features underscores its potential value as a prognostic biomarker. These insights can support improved risk-stratification models and encourage more personalised surveillance intervals for IBD patients. The study also highlights the advantage of integrating such reclassification efforts into daily diagnostic workflows to reduce underdiagnosis. Looking forward, prospective studies combining morphology with molecular and genomic profiling could clarify the biological basis of these dysplasia subtypes. Overall, the findings lay a foundation for more precise diagnostic, prognostic, and therapeutic strategies, promoting earlier intervention and improved outcomes for IBD patients.

## Figures and Tables

**Table 1:** Clinicopathological features of the identified conventional dysplasia subtypes. Abbreviation: CD—Crohn’s disease, IBD—inflammatory bowel disease, TA—tubular adenoma, TVA—tubulovillous adenoma, UC—ulcerative colitis.

	TA (*n* = 106)	TVA (*n* = 24)
**Age at the time of diagnosis**	Average: 61.2 years (median: 63; range: 28–94)	Average: 63.6 years (median: 66; range: 47–82)
**Male: female ratio**	70:36	15:9
**Type of IBD**	UC: 78 (73%) CD: 28 (27%)	UC: 17 (71%) CD: 7 (29%)
**Extent of IBD**	Pancolitis: 31 (29%) Left colon: 53 (50%) Right colon: 22 (21%)	Pancolitis: 8 (33%) Left colon: 13 (54%) Right colon: 3 (13%)
**Duration of IBD**	Average: 14 years (median: 13, range: 1–47)	Average: 13.5 years (median: 12; range: 4–44)
**Localisation of dysplasia**	Left colon: 71 (67%) Right colon: 35 (33%)	Left colon: 17 (71%) Right colon: 7 (29%)
**Grade of dysplasia**	Low-grade: 98 (92%) High-grade: 8 (8%)	Low-grade: 19 (79%) High-grade: 5 (21%)
**Size of dysplasia**	Average: 1.3 cm (median: 1; range: 0.1–2.5)	Average: 1.1 cm (median: 1; range: 0.2–1.6)
**Endoscopic appearance of dysplasia**	Polypoid: 85 (80%) Flat: 21 (20%)	Polypoid: 21 (88%) Flat: 3 (12%)
**Association with other types of conventional dysplasia**	*n* = 7 (7%)	*n* = 9 (38%)
**Association with non-conventional dysplasia**	*n* = 31 (29%)	*n* = 0 (0%)
**Association with adenocarcinoma**	*n* = 24 (23%)	*n* = 2 (8%)

**Table 2:** Clinicopathological features of the identified non-conventional dysplasia subtypes. Abbreviation: CD—Crohn’s disease, GCD—goblet cell-deficient dysplasia, IBD—inflammatory bowel disease, NOS—not otherwise specified, SSL—sessile serrated lesion, TSA—traditional serrated adenoma, UC—ulcerative colitis.

	Serrated dysplasia NOS (*n* = 20)	Hypermucinous dysplasia (*n* = 14)	GCD (*n* = 9)	SSL-like dysplasia (*n* = 6)	TSA-like dysplasia (*n* = 1)
**Age at the time of diagnosis**	Average: 61.3 years (median: 63.5; range: 29–76)	Average: 66.3 years (median: 63.5; range: 43–83)	Average: 61.2 years (median: 63; range: 34–71)	Average: 51.5 years (median: 51; range: 40–56)	70
**Male: female ratio**	9:11	10:4	4:5	5:1	1 male (100%)
**Type of IBD**	UC: 18 (90%) CD: 2 (10%)	UC: 11 (79%) CD: 3 (21%)	UC: 8 (89%) CD: 1 (11%)	UC: 5 (83%) CD: 1 (17%)	UC (100%)
**Extent of IBD**	Pancolitis: 2 (10%) Left colon: 17 (85%) Right colon: 1 (5%)	Pancolitis: 8 (58%) Left colon: 3 (21%) Right colon: 3 (21%)	Pancolitis: 4 (44%) Left colon: 4 (44%) Right colon: 1 (12%)	Left colon: 5 (83%) Right colon: 1 (17%)	Pancolitis (100%)
**Duration of IBD**	Average: 14 years (median: 13; range: 4–40)	Average: 14 years (median: 13; range: 7–28)	Average: 13.7 years (median: 13; range: 3–26)	Average: 15.4 years (median: 16; range: 5–21)	9 years
**Localisation of dysplasia**	Left colon: 9 (45%) Right colon: 11 (55%)	Left colon: 11 (79%) Right colon: 3 (21%)	Left colon: 4 (44%) Right colon: 5 (56%)	Left colon: 4 (67%) Right colon: 2 (33%)	Left colon (100%)
**Grade of dysplasia**	Low-grade: 20 (100%)	Low-grade: 14 (100%)	Low-grade: 9 (100%)	Low-grade: 5 (83%) High-grade: 1 (17%)	Low-grade (100%)
**Size of dysplasia**	Average: 0.53 cm (median: 0.4; range: 0.2–1.2)	Average: 2.25 cm (median: 1.5; range: 0.2–1.6)	Average: 0.8 cm (median: 0.8; range: 0.3–1.4)	Average: 0.8 cm (median: 0.45; range: 1.2–1.1)	0.4 cm
**Endoscopic appearance of dysplasia**	Polypoid: 17 (85%) Flat: 3 (15%)	Polypoid: 9 (64%) Flat: 5 (36%)	Polypoid: 5 (56%) Flat: 4 (44%)	Polypoid: 4 (67%) Flat: 2 (33%)	Flat (100%)
**Association with conventional dysplasia**	*n* = 7 (35%)	*n* = 9 (%)	*n* = 7 (78%)	*n* = 4 (67%)	*n* = 1 (100%)
**Association with other non-conventional dysplasias**	*n* = 5 (25%)	*n* = 4 (%)	*n* = 5 (56%)	*n* = 2 (33%)	*n* = 1 (100%)
**Association with adenocarcinoma**	*n* = 6 (30%)	*n* = 7 (%)	*n* = 7 (78%)	*n* = 6 (100%)	*n* = 0 (0%)

## Data Availability

The data presented in this study are available on request from the corresponding author. The data are not publicly available due to sensitivity concerns and can be shared upon reasonable request.

## Abbreviations

The following abbreviations are used in this manuscript:

CD	Crohn’s disease
CRC	Colorectal cancer
DPD	Dysplasia with increased Paneth cell differentiation
GCD	Goblet cell-deficient dysplasia
HE	Haematoxylin and eosin
IBD	Inflammatory bowel disease
LGD	Low-grade dysplasia
MMR	Mismatch repair
MSI	Microsatellite instability
NOS	Not otherwise specified
OR	Odds ratio
PSC	Primary sclerosing cholangitis
SCENIC	Surveillance for Colorectal Endoscopic Neoplasia Detection and Management in Inflammatory Bowel Disease Patients
SEC	Serrated epithelial change
SSL	Sessile serrated lesion
TA	Tubular adenoma
TED	Terminal epithelial differentiation
TNM	Tumour, lymph node, metastasis
TSA	Traditional serrated adenoma
TVA	Tubulovillous adenoma
UC	Ulcerative colitis
VA	Villous adenoma
WHO	World Health Organisation
